# Efficient micropropagation of *Thunbergia coccinea* Wall. and genetic homogeneity assessment through RAPD and ISSR markers

**DOI:** 10.1038/s41598-022-05787-7

**Published:** 2022-01-31

**Authors:** Kaniz Wahida Sultana, Sumanta Das, Indrani Chandra, Anindita Roy

**Affiliations:** 1 Department of Biotechnology, University of Burdwan, Golapbag, West Bengal 713104 India; 2 Department of Microbiology, M.U.C. Women’s College, Burdwan, West Bengal India

**Keywords:** Biotechnology, Plant sciences

## Abstract

*Thunbergia coccinea* Wall. ex D. Don being a rare, ornamental and medicinal plant of India, is needed to propagate for conserving the germplasm and analyzing its phytochemical compounds in the future. A reliable protocol for direct in vitro propagation using nodal shoot meristem of *T. coccinea* as explant was standardized. The highest number of shoots per explant (22.17 ± 0.54) with maximum shoot length (2.36 ± 0.28) in cm was obtained in Murashige and Skoog (MS) medium supplemented with 9.70 µM of 6-furfurylaminopurine (Kinetin) and 0.053 µM of α-naphthaleneacetic acid (NAA) combination, among all the different plant growth regulators (PGR’s) and concentrations tested. The aforesaid PGR’s combination was optimum for axillary shoot bud induction and multiplication in *T. coccinea*. The best rooting was observed on the half-strength MS medium fortified with 2.68 µM NAA with the highest number of roots per shoot (3.75 ± 0.12) and maximum length (5.22 ± 0.32) in cm. All the in vitro raised plantlets were acclimatized in sterile sand and soil mixture (1:1) with a survival rate of 70% on earthen pots under greenhouse conditions. PCR-based RAPD (Random Amplified Polymorphic DNA) and ISSR (Inter-Simple Sequence Repeat) molecular markers were employed to determine the genetic homogeneity amongst the plantlets. Twelve (12) RAPD and nine (9) ISSR primers developed a total of 104 and 91 scorable bands, respectively. The band profiles of micropropagated plantlets were monomorphic to the mother, donor in vivo plant, and similarity values varied from 0.9542–1.000. The dendrogram generated through UPGMA (unweighted pair group method with arithmetic mean) showed 99% similarities amongst all tested plants confirming the genetic uniformity of in vitro raised plants.

## Introduction

*Thunbergia coccinea* Wall. ex D. Don is a perennial, scarlet red clock vine that belongs to the Acanthaceae family^1^. There is evidence to become rare of this ornamental plant in North-Eastern parts of India^2^. The plant is recorded as a rare medicinal plant in a recent report on the impending threats to medicinal plants of the Himalayas region (North-Eastern India) owing to increased demand^3^. Previous reports have revealed the medicinal importance of the plant as tribal people use the different plant parts to treat fresh wounds, cut, and stomach infections^4,5^. The roots of *T. coccinea* are used to treat tongue blisters and skin infection or inflammation. There is evidence of using the root extract of *T. coccinea* as a health tonic and aphrodisiac in Assam^6^. Antipyretic, analgesic, anti-inflammatory and antioxidant activities of *T. coccinea* leaf reflect its medicinal potency^7,8^. But, the phytochemical constituents of the plant employed in biological activities are still under-explored and the present study might have a great contribution in this discipline. Several phytochemicals like glucosides, phenolic acids and flavonoid compounds have been identified earlier in other species of *Thunbergia* regarding some pharmacological activities. *Thunbergia alata* showed anti-inflammatory, antimicrobial, antiviral and immunomodulatory responses due to presence of iridoid glucosides like thunaloside, alatoside, stilbericoside, 6-epi-stilbericoside, thunbergioside and phenolic acids like caffeoylmalic acid, feruloylmalic acid, p-coumaroylmalic acid^9–11^. *Thunbergia grandiflora* possess two significant iridoid glucosides for example isounedoside having C-10 as a carboxylic acid group and grandifloric acid with a rare 6,7-epoxide functional group. Several reports demonstrated that *T. grandiflora* also contains some important phytochemical constituents including proanthocyanidin (tannin), apigenin-7-glucronide, luteolin (flavonoids) related to antibacterial, antifungal and antihelmentic activities^12,13^. Leaves of *Thunbergia laurifolia* is popular as Thai herbal tea for the high content of glucosides and phenolics including 3'-O-beta-glucopyranosyl stilbericoside, benzyl-2'-O-β-glucopyranosyl glucopyranoside, E-2-hexenyl-β-glucopyranoside, hexanol-β-glucopyranoside, apigenin, apigenin-7-O-β-D-glucopyranoside, chlorogenic acid, 6-C-glucopyranosyl apigenin, 6,8-di-C- glucopyranosyl apigenin^14–16^. In addition, *T. laurifolia* contains gallic acid, caffeic acid, protocatechuic acid, rosmarinic acid and it exhibits antinociceptive, anti-inflammatory, antidiabetic, antidote, detoxification, antitumor, antioxidant and hepatoprotective activities^17–26^. The medicinal importance of *Thunbergia* indicates that the *T. coccinea* might have some significant phytochemicals.

There are many reports of efficient in vitro propagation protocols to conserve and multiply the rare, threatened, endangered plants having ornamental and medicinal values^27^. Being a rare medicinal plant, it is required to propagate on a mass scale. Clonal propagation or micropropagation, an alternative mean of propagation has a significant contribution to cope with insufficiency and further extinction problem facilitating large scale production in a short duration. It can be achieved by direct and indirect organogenesis. Inadequacy of seeds, low germination rate, and unavailability of the fruit of this plant necessitate the establishment of an efficient protocol for in vitro propagation. The previous study was about the indirect regeneration of *Thunbergia coccinea* through somatic embryogenesis from leaf callus^28^, while the present effort is to propagate the plant by direct multiple shoot induction from nodal segments under in vitro conditions. Since there is no report on in vitro propagation of the plant via direct shoot multiplication.

Different abiotic and biotic factors involved in the in vitro process may develop somaclonal variations in regenerated plantlets, particularly in the callus-mediated plantlets^29^. Therefore, the genetic homogeneity assessment of in vitro propagated plantlets is of great importance. Genetic fidelity of regenerants is evaluated through a lot of DNA-based molecular markers like Random Amplified Polymorphic DNA (RAPD), Simple Sequence Repeats (SSR), Inter Simple Sequence Repeat (ISSR) and Amplified Fragment Length Polymorphism (AFLP)^30,31^. RAPD and ISSR are predominant among the various markers for their high reproducibility, reliability, simplicity and cost-effectiveness^32^. The present study includes the assessment of the genetic stability of in vitro propagated plantlets developed through both direct and indirect organogenesis.

## Materials and methods

### Materials for micropropagation

The *Thunbergia coccinea* Wall*.*, a naturally grown plant in the garden of Botany Department, Golapbag, the University of Burdwan (23° 2393ʹ N, 87° 8512ʹ E), West Bengal, India was used as a source of explants. The plant herbarium (Specimen Voucher no. BU/KWS-01) was identified by Kaliyamurthy Karthigeyan, Scientist-E, Central National Herbarium, Botanical Survey of India and deposited at Central National Herbarium, Botanical Survey of India, Howrah, West Bengal. The use of this plant in the present study complies with international, national and/or institutional guidelines. The nodal segments of the plant were used as explants and were surfaced sterilized with 70% (v/v) ethanol for 30 s, followed by washing with 0.1% (v/v) tween 20 solution and then the explants were treated with 0.1% mercuric chloride (HgCl_2_) solution for 1.0–1.5 min followed by the rinsing with sterile dH_2_O for three times.

### Culture medium and growth conditions

After cutting the ends of the nodal segments, explants were inoculated on the Murashige and Skoog basal media (Hi-media, India) supplemented with 0.44 g^−1^ CaCl_2_ (Hi-media, India), 30 gL^−1^ sucrose (Hi-media, India), 1.5 gL^−1^ phytagel (Sigma Aldrich, USA), and different concentrations of plant growth regulators (PGRs) like naphthalene acetic acid (NAA) (Sigma Aldrich, USA), 6-benzylaminopurine (BAP) (Sigma Aldrich, USA), 6-furfurylaminopurine (Kin) (Sigma Aldrich, USA), thidiazuron (TDZ) (Sigma Aldrich, USA). The pH of the medium was adjusted to 5.8 ± 0.02 before autoclaving at 121 °C for 15 min^33^. The cultures were incubated in a plant growth chamber at 25 ± 2 °C temperature with 55% humidity, 16/8 h (light/dark cycle) of photoperiod provided with white fluorescent light of 2000 lx intensity.

### PGRs treatments for direct shoot induction establishment and its proliferation

The effect of different concentrations and combinations of PGRs in the fortified MS media was studied on the shoot bud initiation, shoot multiplication and shoot elongation. Treatments with PGRs like BAP of 4.44 µM, 8.88 µM and 13.32 µM, Kin of 4.85 µM, 9.70 µM and 14.55 µM, and TDZ of 4.55 µM and 9.10 µM, in combination with NAA of 0.053 µM, 0.53 µM or individually were tested to establish and standardize the maximum number of shoot multiplication. Each treatment including control was done in triplicates and after 40 d of incubation, the percentage of shoot induction, number of shoots per explants, and shoot length (cm) were recorded.

### Rooting

The individual shoot was transferred to the full strength and half-strength MS media supplemented with NAA of 0.53 µM, 2.68 µM and 5.37 µM after cutting off the multiplied shoots. The percentage of root induction and root numbers per shoot were recorded after 40 d.

### Acclimatization

Regenerated plantlets were hardened in polythene bags containing sterile soil and sand mixture (1:1) after rinsing the plantlets with sterile water to wash of adhering medium residue and then covered with another polythene bags to maintain high humidity. The plants were kept in the greenhouse at 25 ± 2 °C temperature with 75–85% humidity. After hardening for 15 d, the cover of the polythene bags was removed, and then after 10 d, the plants were transferred to earthen pots filled with garden soil for acclimatization under greenhouse conditions.

### Assessment of genetic homogeneity by RAPD and ISSR

Leaves from selected in vitro plantlets and mother plant (in vivo* T. coccinea*) were used for the extraction of genomic DNA by the cetyltrimethylammonium bromide (CTAB) method^34^. Quantification of DNA was accomplished by analyzing the DNA on 1% agarose gel using diluted uncut λ (lambda) DNA as a standard. Finally, all the genomic DNA samples were diluted to a final concentration of 40 ng/µl with 1X TE buffer (10 mM Tris–HCl; pH 8.0; 1 mM EDTA). DNA samples were stored at − 20 °C for further use. A set of twelve (12) RAPD primers and a set of nine (9) ISSR primers have been used to evaluate the polymorphism among the in vitro grown plantlets including callus-mediated plantlets and mother plants. PCR amplification reactions were carried out in a 20 µl cocktail containing 40 ng of genomic DNA template, 1X buffer, 1 µl of Taq DNA polymerase, 1.5 mM MgCl_2_, 2.5 mM dNTPs, 10 mg/ml BSA and 10 pmol primers. The PCR amplification protocol was programmed in a thermal cycler (Applied Biosystems Corp., USA) for reaction steps of an initial denaturation at 94 °C for 5 min, 38 cycles of 94 °C for 30 s, primer annealing 30–55 °C for 30 s, extension at 72 °C for 1 min, 30 s final extension step of 72 °C for 5 min. The PCR products were analyzed on 1.8% agarose gel along with 1000 bp molecular weight marker for RAPD and photographed under UV transilluminator using Bio-Rad documentation gel system (Bio-Rad Laboratories Inc., USA). The banding patterns generated by RAPD and ISSR analysis were scored to determine the genetic variance among tested samples. The data matrix was prepared based on the presence and absence of amplified fragments as 1 and 0, respectively. Jaccard’s coefficient was used to estimate the genetic similarity and the similarity matrix was used in the cluster analysis which was performed with NTSYSpc 2.2 software using unweighted pair group method with arithmetic mean (UPGMA)^35,36^.

### Statistical analysis

Data from all experiments were analyzed with SPSS 26.0 version software package (SPSS Inc., USA) to measure the mean using one-way ANOVA. Duncan’s Multiple Range Test was carried out to compare and determine the significant difference in means at 5% probability level (P ≤ 0.05)^37^.

## Results and discussion

### Effect of cytokinins on shoot bud induction and shoot bud multiplication

**Figure 1:**
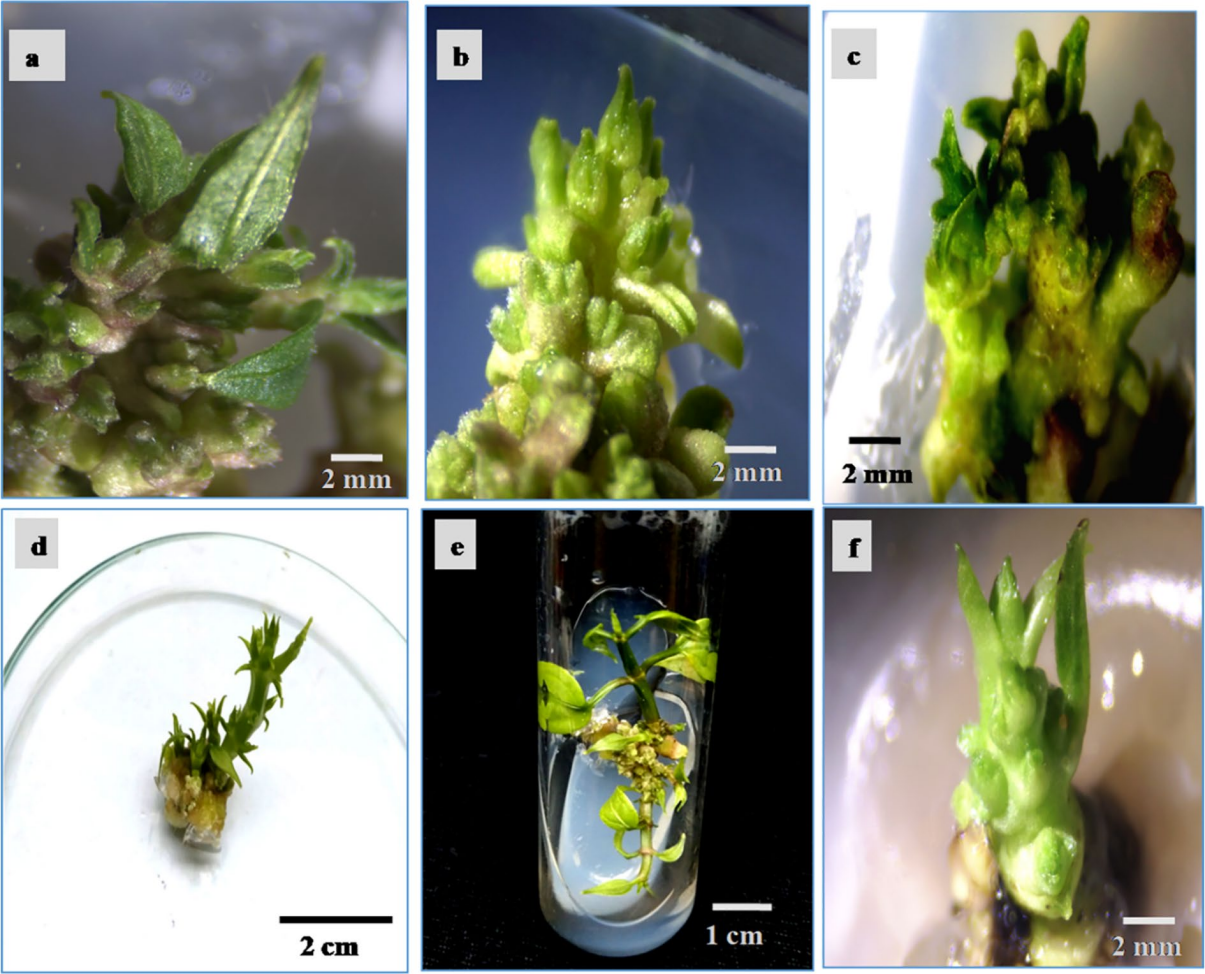
Effect of BAP, Kin, TDZ and NAA on shoot induction and shoot multiplication from nodal meristem of *T. coccinea*. (**a**) Axillary shoot buds induced on NAA 0.053 µM + Kin 9.70 µM after 10 d of inoculation; (**b,c**) Axillary shoot buds induced on NAA 0.053 µM + BAP 8.88 µM with basal callus after 10 d; (**d**) Proliferated shoots with a basal callus on BAP with NAA after 40 d of inoculation; (**e**) Proliferated shoots on Kin 9.70 µM with NAA 0.053 µM; (**f**) Shoots with callus on TDZ 4.55 µM along with Kin 4.85 µM.

Several reports on in vitro micropropagation of medicinal plant species such as *Momordica dioica*, *Passiflora foetida*, *Salvadora persica,* etc. suggested the use of nodal explants (nodal meristem) for the presence of cytokinins at the nodal region resulting in activation of axillary buds^38,39^. In the present study, nodal segments of the plant were inoculated into the shoot induction media following sterilization and then growth was initiated after 10 d. The axillary buds had appeared after 10–20 d of inoculation on the shoot induction media. Nodal explants on MS medium without any cytokinin showed 10% response for axillary bud induction. The highest frequency of shoot bud induction (83%) was noticed on BAP of 8.88 µM as well as Kin of 9.70 µM in combination with 0.053 µM NAA as mentioned in Table 1. Though, both BAP and Kin showed their effectiveness in the induction of lateral shoot buds (Fig. 1a,b), but inevitable basal callus was induced in the treatments containing BAP alone and in combinations with NAA (Fig. 1c,d). TDZ alone and in combination with Kin showed no significant difference in the frequency of shoot bud induction after 10 d of inoculation (Table 1). The rate of shoot proliferation was increased with increasing concentration of BAP and Kin beyond the optimum level it declined in *T. coccinea*. The result was substantiated by the early report claiming that the rate of shoot proliferation from nodal meristem is increased with increasing concentrations of cytokinins to some extent^39^.

**Table 1:** Frequency of shoot bud induction in response to different concentrations and combinations of NAA, BAP, Kin and TDZ after 10 d of inoculation from the nodal meristem.

Plant growth regulators (µM)				Frequency of shoot bud induction after 10 days (%)	Description
NAA	BAP	Kin	TDZ		
0.0	0.0	0.0	0.0	10	Small shoot buds
0.053	4.44	0.0	0.0	62	Small buds, callus
0.053	8.88	0.0	0.0	83	Dense buds, callus
0.053	13.32	0.0	0.0	65	Small buds, callus
0.53	4.44	0.0	0.0	60	Buds, callus
0.53	8.88	0.0	0.0	70	Shoot buds, callus
0.53	13.32	0.0	0.0	50	Shoot buds, callus
0.053	0.0	4.85	0.0	65	Dense buds, no callus
0.053	0.0	9.70	0.0	83	Dense buds, no callus
0.053	0.0	14.55	0.0	74	Dense buds, no callus
0.53	0.0	4.85	0.0	60	Shoot buds, no callus
0.53	0.0	9.70	0.0	70	Dense buds, no callus
0.53	0.0	14.55	0.0	70	Dense buds, no callus
0.0	4.44	0.0	0.0	55	Shoot buds, callus
0.0	8.88	0.0	0.0	75	Shoot buds, callus
0.0	13.32	0.0	0.0	61	Shoot buds, callus
0.0	0.0	4.85	0.0	70	Shoot buds, no callus
0.0	0.0	9.70	0.0	74	Shoot buds, no callus
0.0	0.0	14.55	0.0	60	Shoot buds, no callus
0.0	0.0	0.0	4.55	40	Shoot buds, no callus
0.0	0.0	0.0	9.10	65	Shoot buds, no callus
0.0	0.0	4.85	4.55	45	Shoot buds, hyperhydricity, callus
0.0	0.0	4.85	9.10	30	Shoot buds, hyperhydricity, callus
0.0	0.0	9.70	4.55	55	Shoot buds, callus
0.0	0.0	9.70	9.10	35	Shoot buds, callus

**Figure 2:**
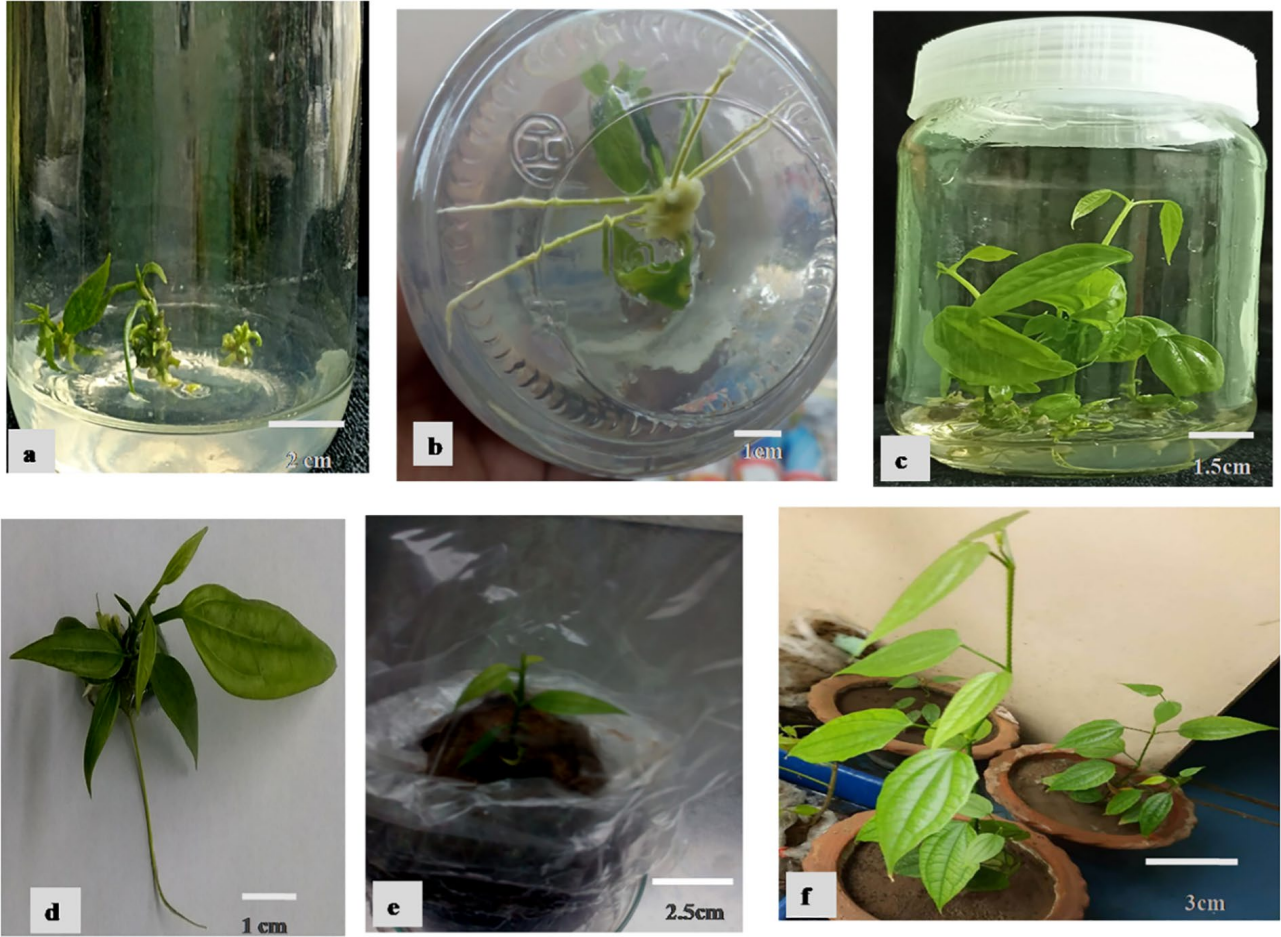
Shoot multiplication, rooting, and acclimatization (**a**) Shoots on Kin 9.70 µM with NAA 0.053 µM; (**b**) Rooting of regenerated shootlets on half-strength MS medium supplemented with NAA 2.68 µM; (**c,d**) In vitro plantlets; (**e**) Hardening of plantlets in sand and soil mixture (1:1) covered with polythene bags. (**f**) Acclimatized plants on earthen pot soil in the greenhouse.

The intervening callus induced on shoot multiplication media containing BAP and NAA decreased the number of shoots and shoot length in *Vigna radiate*^40^. In line with the aforementioned report, the short and reduced number of shoots per explants of *T. coccinea* was produced with BAP along with NAA in presence of intervening callus as depicted in Fig. 1d and BAP alone was also insignificant to shoot multiplication as shown in Fig. 1c. Whereas, the effect of Kin along with NAA was better in shoot multiplication and elongation of *T. coccinea* than BAP accompanying NAA. Among all the treatments, Kin gave rise to the highest number of shoots per explant i.e., 22.17 ± 0.54 (Table 2 and Fig. 1e) and the significantly highest shoot length was achieved in these combinations of PGRs (Fig. 2a). The shoot length on MS medium fortified with Kin 9.70 µM and NAA 0.053 µM was 2.36 ± 0.28. The shoot numbers were decreased with the increased concentration of NAA along with Kin. Adugna et al. reported a similar effect of kinetin along with a low concentration of NAA on shoot multiplication of *Moringa stenopetala*^41^. It was observed that BAP 8.88 µM combined with NAA 0.053 µM produced the second maximum mean number of shoots per explants (15.30 ± 0.33). The mean length of shoot was significantly different i.e., 1.55 ± 0.24 on BAP 8.88 µM and NAA 0.053 µM combination, but 1.5 times less than shoot length on Kin 9.70 µM and NAA 0.053 µM as reflected in Table 2.

**Table 2:** Effect of different concentrations and combinations of NAA, BAP, Kin and TDZ on shoot multiplication after 40 d of inoculation.

Plant growth regulators (µM)				No. of shoots per explant (Mean ± SE) after 40 d	Mean of shoot length (cm) (Mean ± SE) after 40 d
NAA	BAP	Kin	TDZ		
0.0	0.0	0.0	0.0	2.33 ± 0.21^ h^	0.31 ± 0.25^gh^
0.053	4.44	0.0	0.0	9.27 ± 0.37^cde^	0.49 ± 0.10^ef^
0.053	8.88	0.0	0.0	15.30 ± 0.33^b^	1.15 ± 0.30^bc^
0.053	13.32	0.0	0.0	7.25 ± 0.25 ^def^	0.50 ± 0.23^ef^-
0.53	4.44	0.0	0.0	5.25 ± 0.66^ fg^	0.34 ± 0.12^gh^
0.53	8.88	0.0	0.0	8.00 ± 0.65 ^def^	0.77 ± 0.15^ cd^
0.53	13.32	0.0	0.0	7.90 ± 0.34 ^def^	0.53 ± 0.03^ef^
0.053	0.0	4.85	0.0	10.75 ± 0.55^ cd^	0.75 ± 0.04^ cd^
0.053	0.0	9.70	0.0	22.17 ± 0.54^a^	2.36 ± 0.28^a^
0.053	0.0	14.55	0.0	12.25 ± 0.15^ cd^	0.73 ± 0.13^ cd^
0.53	0.0	4.85	0.0	5.45 ± 0.42^efg^	0.37 ± 0.02^gh^
0.53	0.0	9.70	0.0	9.80 ± 0.47^cde^	0.78 ± 0.02 cd
0.53	0.0	14.55	0.0	7.55 ± 0.54 ^def^	0.81 ± 0.02^c^
0.0	4.44	0.0	0.0	12.20 ± 0.67 cd	0.55 ± 0.05^ef^
0.0	8.88	0.0	0.0	9.65 ± 0.17^cde^	0.65 ± 0.24^de^
0.0	13.32	0.0	0.0	7.20 ± 0.36 ^def^	0.68 ± 0.02^de^
0.0	0.0	4.85	0.0	11.75 ± 0.55^ cd^	0.69 ± 0.57^de^
0.0	0.0	9.70	0.0	12.50 ± 0.54^ cd^	1.55 ± 0.24^b^
0.0	0.0	14.55	0.0	6.05 ± 0.42^ fg^	0.77 ± 0.45^ cd^
0.0	0.0	0.0	4.55	7.61 ± 0.33 ^def^	0.29 ± 0.15^gh^
0.0	0.0	0.0	9.10	4.10 ± 0.21^gh^	0.84 ± 0.08^c^
0.0	0.0	4.85	4.55	10.45 ± 0.78 cd	0.33 ± 0.09^fgh^
0.0	0.0	4.85	9.10	5.70 ± 0.60^efg^	0.21 ± 0.53^ h^
0.0	0.0	9.70	4.55	5.76 ± 0.40^efg^	0.45 ± 0.10^fgh^
0.0	0.0	9.70	9.10	6.30 ± 0.57^efg^	0.32 ± 0.08^fgh^

Each value represents the mean ± SE of three replicates for the number of shoots proliferated per explant and shoot length. Different letters in the same column indicate the significant mean difference at P ≤ 0.05 (Duncan’s multiple range test).

There was no significant difference in the mean of shoot number with treatments like BAP, Kin and TDZ alone for shoot multiplication and elongation (Table 2). In the present study, it was found that NAA of 0.53 µM along with BAP induced profuse callus (Fig. 1d) and it retarded the growth of shoots. Therefore, a high cytokinin to very low auxin ratio was optimum for shoot multiplication. Cytokinins have a great contribution to plant tissue culture for its cell differentiation and cell elongation activities. Shoot proliferation is the major function of cytokinin, but the synergistic balance between the cytokinin and auxin regulates the consistent and successful growth of shoots^42^. According to Song et al. (2010), a combination of cytokinin and an auxin is often used to achieve high ratios of shoot induction^43^. Therefore, the effect of BAP and Kin were tested in combination with a low concentration of NAA in the present experiment. As the early reports suggested the greater shoot proliferation in TDZ at low concentration alone and in combination with cytokinin^44^, shoot proliferation and elongation were also studied with TDZ in the *T. coccinea*. But there was no improvement in shoot multiplication rather it induced callus with hyperhydric shoots (Fig. 1f). Some reports about the effect of TDZ on species like *Cercis canadensis*, *Vitis rotundifolia* and *Pyrus pyrifolia* corroborated the present findings^45^. Varied translocation rates of cytokinins to the responsive regions, their differential uptake, varied effect on metabolic processes and ability to change the level of endogenous cytokinins may influence the response with different cytokinins and different explants during in vitro propagation^46^.

### Rooting

In vitro regenerated shootlets showed no response in MS medium without any growth regulators. The regenerated shootlets were inoculated on half and full-strength MS medium supplemented with NAA (0.26–5.37 µM) to induce roots. The best result for rooting was recorded in a half-strength MS medium containing 2.68 µM NAA after 40 d of inoculation (Fig. 2b) and a maximum of 3.75 ± 0.12 roots per shoot with 5.22 ± 0.32 cm root length was induced on the medium (Table 3). Earlier researchers established the beneficial effect of reducing the concentration of MS basal medium on in vitro rooting in *Quercus sobur* L., *Solanum trilobatum* and *Wrightia tomentosa*^47–49^. Half strength MS basal medium suited the best for in vitro rooting in regenerated *T. coccinea* shootlet*s*. Many reports found the effectiveness of NAA on rooting in plant species like *Iris sanguinea*, *Scaevola serica* and *Withania somnifera* which is consistent with the present outcome of *T. coccinea*^50–52^.

**Table 3:** Effect of different concentrations of NAA on rooting with half strength and full-strength MS medium.

Medium	Plant growth regulator (µM)	Frequency of rooting (%)	No. of roots per explant (Mean ± SE)	Mean of root length (cm) (Mean ± SE)	Description
	**NAA**				
½ strength MS	0.00	00	0.00 ± 0.00^ g^	0.00 ± 0.00^ h^	No response
	0.26	50	1.50 ± 0.25f.	1.15 ± 0.47^ fg^	Lateral roots
	0.53	72	1.55 ± 0.35^def^	1.22 ± 0.45^ fg^	Lateral roots
	2.68	91	3.75 ± 0.12^a^	5.22 ± 0.32^a^	Lateral roots with insignificant callus
	5.37	85	3.25 ± 0.12^b^	3.61 ± 0.37^bc^	Lateral roots with insignificant callus
Full strength MS	0.00	00	0.00 ± 0.00^ g^	0.00 ± 0.00^ h^	No response
	0.26	72	3.21 ± 0.36^bc^	3.24 ± 0.40^bc^	Lateral roots with callus
	0.53	60	1.75 ± 0.20^de^	1.47 ± 0.33f.	Callus with a few short root
	2.68	52	2.33 ± 0.40^bcd^	1.14 ± 0.35^ fg^	Callus with a few short root
	5.37	48	2.15 ± 0.^bcd^	1.00 ± 0.15^ g^	Callus with a few short root

Each value represents the mean ± SE of three replicates for the number of roots per explant and root length. Different letters in the same column indicate the significant mean difference at P ≤ 0.05 (Duncan’s multiple range test).

### Acclimatization

The acclimatization of in vitro regenerated plantlets was a difficult step of the micropropagation protocol establishment of their susceptibility to fungal diseases^53^. In the present study, the healthy rooted plantlets (Fig. 2c,d) were transferred to sterile soil and sand mixture (1:1) followed by rinsing thoroughly with sterile water. A similar combination of 1:1 ratio of compost and soil was followed for successful hardening of *Morus* spp.^54^. The plantlets must be watered and covered within polythene sheets to maintain high humidity (Fig. 2e). A diluted carbendazim solution was sprayed to prevent fungal infection and to increase its tolerance to environmental stresses. The polythene sheets were pricked for proper ventilation and after 15 d, the polythene covers were removed to increase their survival rate in the environmental conditions followed by transferring those plantlets to the greenhouse (Fig. 2f). The survival rate for the regenerated plantlets was increased to 70% from the previous study of *T. coccinea*^28^.

### Genetic homogeneity analysis with RAPD and ISSR

It is necessary to assess the genetic stability among the in vitro raised plantlets and mother plants (in vivo plant) for the establishment of a micropropagation protocol. Tables 4 and 5 revealed the results of 4 different samples of *T. coccinea* represented as TC1 (mother plant), TC2 (in vitro raised direct regenerants), TC3 (in vitro raised direct regenerants), and TC4 (callus mediated plants). In the present study, a previously reported callus-derived *T. coccinea* regenerant sample (TC4) was also assessed with the mother and direct propagated plants to check their genetic variability using RAPD and ISSR markers. Authors adopted the two PCR-based RAPD and ISSR analyses amongst the various molecular techniques due to their ease of use, cost and time effectiveness.

**Table 4:** List of RAPD primers, their sequences, number of scorable bands and their range of amplified fragments generated in *T. coccinea* mother plant and regenerants.

Primer code	Sequence (5´-3´)	Number of scorable bands	Number of bands		Approximate range of amplification (bp)
			Monomorphic	Polymorphic	
B18	GAGAGCCAAC	8	8	0	200–1000
OPC5	GATGACCGCC	6	6	0	100–1000
OPL12	GGGCGGTACT	4	4	0	400–900
OPA15	TGCCGAGCTA	7	7	0	300–1000
OPA18	AGGTGACCGT	11	10	1	250–1200
OPD 3	GTCGCCGTCA	11	11	0	100–1100
OPV5	TCCGAGAGGG	12	12	0	150–1200
OPV14	AGATCCCGCC	10	7	2	200–1000
OPV2	AGTCACCCC	5	5	0	250–1000
OPY 4	GGCTGCAATG	8	8	0	200–1000
OPZ1	TCGGATCCGT	12	12	0	100–1200
OPW19	CAAAGCGCTC	10	10	0	150–1000
Total		104	101	3

**Table 5:** List of RAPD primers, their sequences, number of scorable bands and their range of amplified fragments generated in *T. coccinea* mother plant and regenerants.

Primer code	Sequence (5´- 3´)	No. of scorable bands	No. of bands		Approximate range of amplification (bp)
			Monomorphic	Polymorphic	
UBC 807	AGAGAGAGAGAGAGAGT	13	13	0	100–1500
UBC 812	GAGAGAGAGAGAGAGAA	10	10	0	100–1300
UBC 815	CTCTCTCTCTCTCTCTG	11	11	0	250–1500
UBC 820	GTGTGTGTGTGTGTGTC	9	8	2	500–1800
UBC 822	TCTCTCTCTCTCTCTCA	9	9	0	400–1700
UBC 827	ACACACACACACACACG	9	9	0	300–1500
UBC 846	CACACACACACACACAAT	14	14	1	100–1700
UBC 847	CACACACACACACACARC	11	11	0	350–1800
B17898	CACACACACACAGT	14	14	0	100–1800
Total		91	88	3	

### RAPD analysis

**Figure 3:**
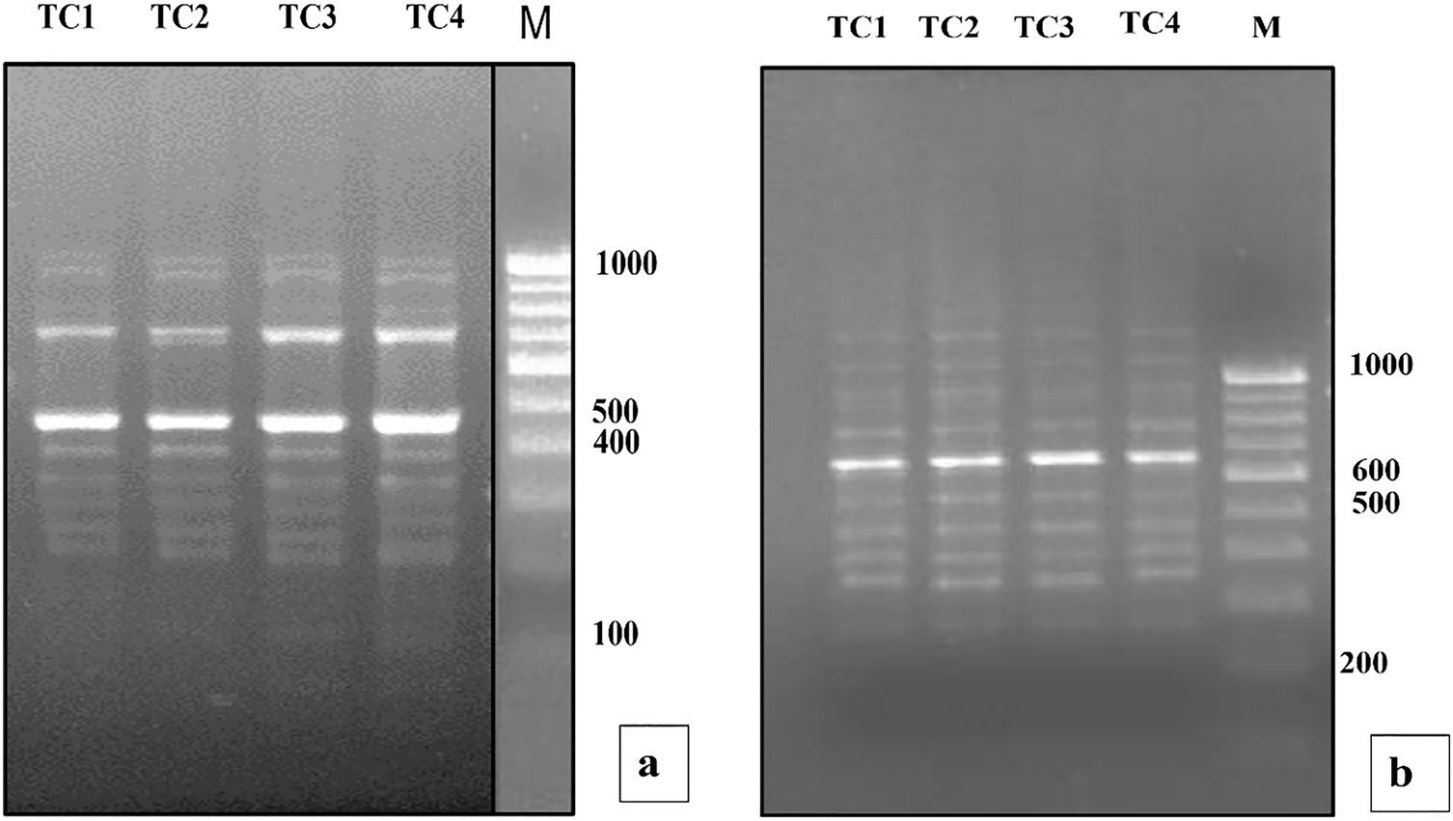
RAPD profiles generated by PCR amplification with primer (**a**) OPC 5 and (**b**) OPA 15. Lane **M**: Molecular marker (100–1500 bp); Lane **TC 1**: In vivo mother plant; Lane **TC2-3**: In vitro propagated plantlets; **TC 4**: Callus-derived plantlets. (The full-length gels/blots are presented in Supplementary Fig. 1a,b).

12 RAPD primers generated a total of 104 distinct and scorable bands with an average of 8.6 bands per primer with sizes ranging from 100–1200 bp. All the bands of in vitro raised plants were monomorphic to the mother plant (TC1) with the RAPD primers except OPA18 and OPC14 which displayed only 3 polymorphic bands in TC3 and TC4 (Table 4). Monomorphism among all the regenerants and mother plants with the RAPD primers such as OPC5 and OPA15 was visualized to confirm the genetic uniformity and stability of the regenerants of *T. coccinea* (Fig. 3).

### ISSR analysis

**Figure 4:**
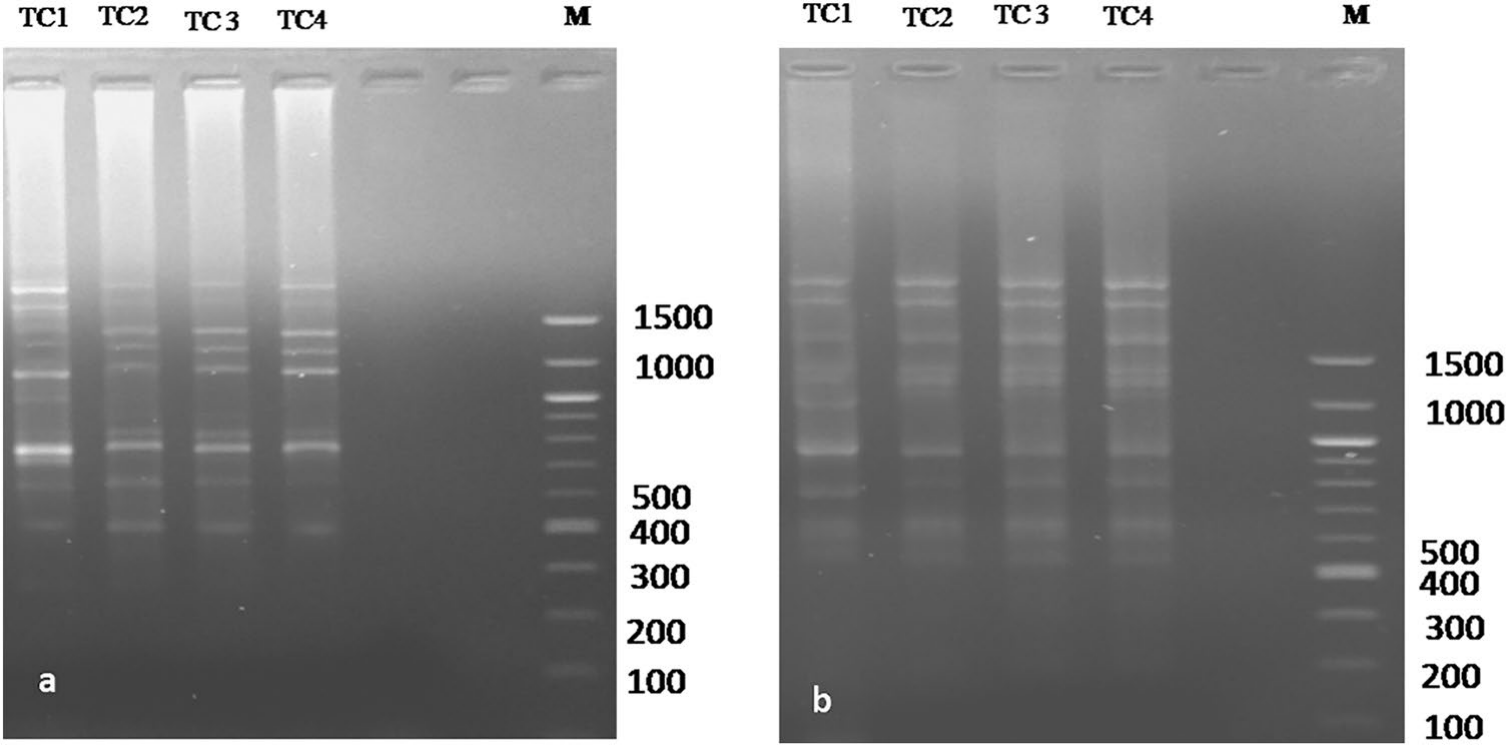
ISSR profiles generated by PCR amplification with primer (**a**) UBC822 and (**b**) UBC 846. Lane **M**: Molecular marker (100–1500 bp); Lane **TC 1**: In vivo mother plant; Lane **TC2-3**: In vitro propagated plantlets; **TC 4**: callus-derived plantlets.

In the case of ISSR analysis, 9 ISSR primers developed a total of 91 distinct and scorable bands with an average of 10.1 bands per primer ranging in size from 100–1800 bp (Table 5). Monomorphism among the mother plant (TC1) and three in vitro r﻿﻿egenerants of *T. coccinea* were detected by the 7 ISSR primers (Fig. 4). Three polymorphic bands were observed in only TC4 regenerants with two UBC primers like UBC 820 and UBC 846.

### Dendrogram

**Figure 5:**
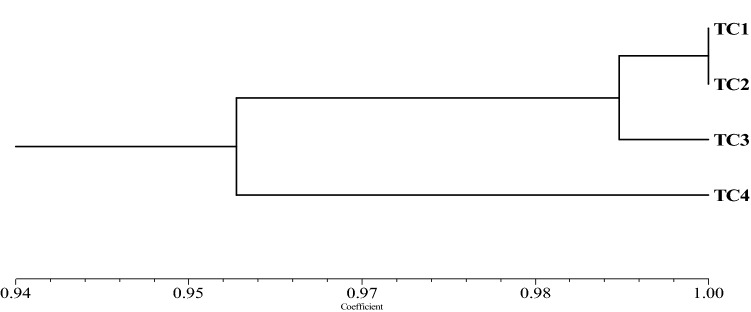
UPGMA dendrogram showing genetic relationship among the regenerants of *T. coccinea* and their mother plant based on Jaccard’s similarity indices from RAPD and ISSR data.

The similarity indices were estimated from the combined data of RAPD and ISSR using Jaccard’s similarity coefficient between the in vitro raised plants and their mother plant ranging from 0.9542–1.000. The UPGMA analysis grouped all 4 genotypes into two major groups at a similarity coefficient of 0.9542 indicating the low genetic variations among the mother plant and in vitro regenerants. One major group includes the mother plant and two in vitro direct propagated plants through axillary bud proliferation and the other group includes callus mediated plant (Fig. 4). TC1 and TC2 plants showed maximum genetic similarity between them with a similarity coefficient of 1.000, while TC1 and TC2 showed genetic similarity with TC3 and TC4 with a similarity coefficient of 0.9922 and 0.9615 respectively. But, the similarity coefficient between TC3 and TC4 was 0.9542 (Table 6). Jaccard’s similarity indices measure the genetic distance between the tested samples. TC1, TC2 and TC3 were pretty closed to each other, whereas, the distances of TC4 to TC1, TC2 and TC3 were 0.04. Therefore, the UPGMA analysis confirmed the genetic stability and uniformity amongst the mother plant and in vitro propagated plants with a very low percentage (1%) of variation as indicated in Fig. 5. Overall, all the in vitro raised plants of *T. coccinea* including callus mediated plants were genetically stable. Naturally occurring variations including environmental factors, accumulation of mutation by factors like duration of treatment, in vitro stress, auxin to cytokinin ratio (hormonal balance), added biochemicals, nutritional conditions, all of which played a vital role in the development of small genetic variation^55^. On contrary to the reports of no genetic variation among the micropropagated plant and mother plant in *Asparagus officinalis*, *Chlorophytum arundinaceum*, *Simmondsia chinensis*^56–58^, there are some reports of somaclonal variation in *Codonopsis lanceolata* Benth et Hook, *Dactyospermum ovalifolium* Wight, *Spilanthes calva*, *Jatropha curcas* developed during in vitro micropropagation^29,59,60^. As the callus incurred genetic variation in the callus-mediated plants, the protocol of micropropagation through direct shoot proliferation for the *T. coccinea* demonstrated by the author in the present study was established successfully. The axillary shoot proliferation minimizes the chance of variability in the in vitro plants, consistent with the previous reports^38,61,62^.

**Table 6:** Similarity matrices of the 3 micropropagated plants of *T. coccinea* and their corresponding mother plant based on Jaccard’s similarity coefficient from RAPD and ISSR markers.

	TC1	TC2	TC3	TC4
TC1	1.000			
TC2	1.000	1.000		
TC3	0.9922	0.9922	1.000	
TC4	0.9615	0.9615	0.9542	1.000

## Conclusion

In summary, this work establishes an efficient protocol for the micropropagation from the nodal shoot meristem of *Thunbergia coccinea* through axillary bud multiplication in contrast to the reported study of the authors was about the callus induction and indirect regeneration of *T. coccinea*. Maximum shoot induction and shoot multiplication was achieved on MS medium containing 9.70 µM of Kin along with 0.053 µM of NAA for direct regeneration of the plant. The highest number of roots with maximum length was observed on half strength MS medium supplemented with 2.68 µM of NAA. The experimental findings of genetic homogeneity testing through RAPD and ISSR markers among the mother plant and all in vitro raised plants strongly suggest that the risk of genetic instability can be reduced with direct axillary shoot proliferation. Hence, this protocol may be useful for the commercial multiplication of *T. coccinea*. Moreover, the findings will play a significant role to meet the demand of this plant and it will also provide support to the researcher for phytochemical analysis.

## Supplementary Information

Supplementary Information.
