# Supplementary material for: Efficient micropropagation of Thunbergia coccinea Wall. and genetic homogeneity assessment through RAPD and ISSR markers
Source: Sci Rep. 2022 Jan 31;12:1683. doi: 10.1038/s41598-022-05787-7 (PMC8803877; doi:10.1038/s41598-022-05787-7)
Supplement: Supplementary file 1 — Supplementary Information. [file 41598_2022_5787_MOESM1_ESM.docx]

## **Efficient micropropagation of *Thunbergia coccinea* Wall. and genetic homogeneity assessment through RAPD and ISSR markers**

Kaniz Wahida Sultana^1^, Sumanta Das^1^, Indrani Chandra^1*^, and Anindita Roy^2^

^*^Corresponding author e-mail id- ichandrabiotech@gmail.com

^1^Department of Biotechnology, University of Burdwan, Golapbag, West Bengal, India, 713104

^2^Department of Microbiology, M.U.C. Women’s College, Burdwan, West Bengal, India

**Supplementary picture**

**Figure 1 (S)**


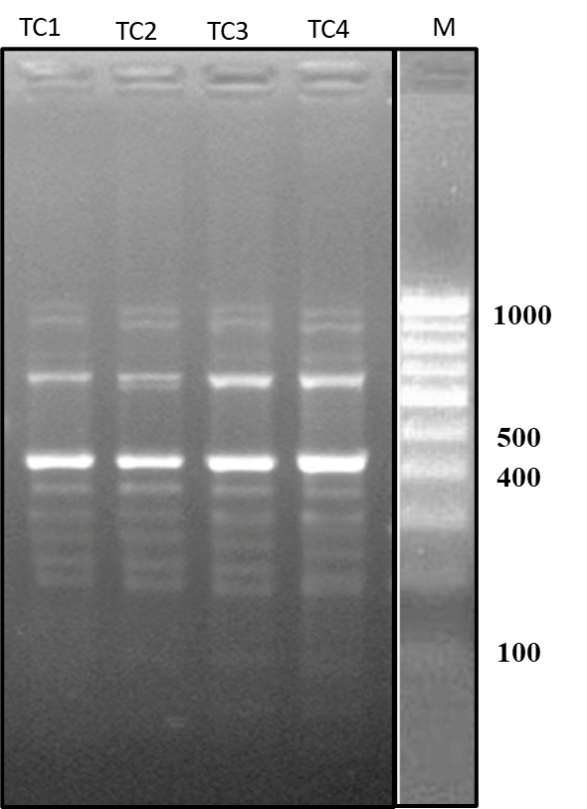


**Figure 1(S) a.** RAPD profiles generated by PCR amplification with primer OPC 5 and. Lane **M**: Molecular marker (100–1500 bp); Lane **TC 1**: In vivo mother plant; Lane **TC2-3**: In vitro propagated plantlets; **TC 4**: Callus-derived plantlets


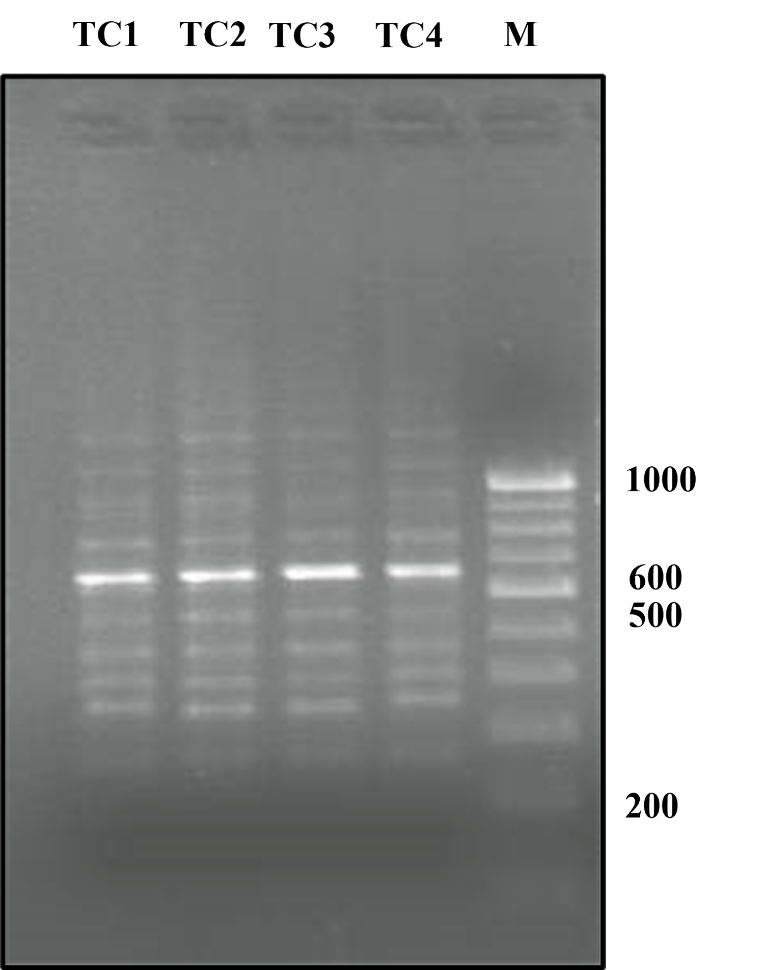


**Figure 1(S) b.** RAPD profiles generated by PCR amplification with primer OPA 15 Lane **M**: Molecular marker (100–1500 bp); Lane **TC 1**: In vivo mother plant; Lane **TC2-3**: In vitro propagated plantlets; **TC 4**: Callus-derived plantlets
